# Recent advances and public health implications for environmental exposure to *Chlamydia abortus*: from enzootic to zoonotic disease

**DOI:** 10.1186/s13567-022-01052-x

**Published:** 2022-05-31

**Authors:** Lauretta Turin, Sara Surini, Nick Wheelhouse, Mara Silvia Rocchi

**Affiliations:** 1 Department of Veterinary Medicine (DIMEVET), University of Milan, Milan, Italy; 2 School of Applied Sciences, Edinburgh Napier University, Sighthill Court, Edinburgh, EH11 4BN UK; 3 Moredun Research Institute, Bush Loan, Pentlands Science Park, Penicuik, EH26 0PZ Scotland, UK

**Keywords:** *Chlamydia abortus*, public health, environmental exposure, interspecies transmission

## Abstract

Environmental transmission of *Chlamydia abortus* as a result of enzootic disease or disease outbreaks and the threats posed by this pathogen has been previously reported, however a state-of-the-science review of these reports and the identification of future research priorities in this area is still lacking. This study provides an overview of the current knowledge of host–pathogen–environment interactions, addressing public health risks and identifying critical questions and research gaps. We performed a systematic PubMed and Web of Science search for publications related to *Chlamydia abortus* in the past four decades, and we reviewed and combined the evidence critically discussing and commenting the results. A total of 182 studies, 5 chapters of specific books and the “OIE terrestrial manual” were included in this review. There were substantial variations between the studies in topic addressed and experimental design. Overall, the literature largely supports the crucial role played by environmental exposure on the acquisition of zoonotic disease caused by *Chlamydia abortus*. We also identify the paucity of information related to interspecies transmission and pathogen adaptation in relation to environmental dissemination and zoonotic risk. This analysis further highlights the need for additional research given that environmental transmission represents a serious risk not only to susceptible patients (pregnant women and immunocompromised individuals), but also for other species including wildlife.

**Supplementary Information:**

The online version contains supplementary material available at 10.1186/s13567-022-01052-x.

## Introduction

*Chlamydia abortus* (*C. abortus*) is a non-motile obligate intracellular Gram-negative pathogenic bacterium, belonging to the *Chlamydiales* family. *C. abortus* infects mainly ruminants, especially sheep and goats, and less frequently cattle, pigs and horses; however, it can also affect humans, being of particular concern in pregnant women [1, 2]. *C. abortus* is known as the causative agent of enzootic abortion of ewes (EAE) or ovine enzootic abortion (OEA) which represents one of the most common causes of ovine and caprine infectious abortion worldwide, along with other infectious agents such as *Campylobacter* sp, *Toxoplasma* sp, *Listeria* sp, *Salmonella* sp, Border disease virus and Cache Valley virus [3, 4]. Abortion occurs in the later stages of pregnancy, as *C. abortus* is able to progressively colonize the placenta, causing damage and affecting the fetus(es) to varies degrees [5]. The infection can result in foetal loss (abortion), the birth of stillborn or weak lambs or, in some cases of unaffected animals; presence of a weak lamb with a healthy tween is also not uncommon [6]. Breeders can incur great economic losses if numerous cases occur in a farm (abortion storm), usually when the infection first affects a naïve flock [7]. Another important aspect is the spread of this enzootic infection to humans, which can develop as severe disease, especially in pregnant women [2], generally affecting female farmers, abattoir workers and veterinarians. However, environmental contamination with the bacteria released by abortion products or infected animals may also play a crucial role in disease spread, interspecies cross-over and adaptation [8]. Indeed, abortion products, in particular vaginal fluids, placentas, dead/aborted lambs, fleeces and still born/infected lambs are all characterized by a high bacterial load and represent a significant risk, both for naïve animals and for humans [9]. Different types of flock management are also involved in the extent of environmental contamination and the spread of the pathogen: in intensively managed flocks, where the animals are kept in smaller enclosures, there is a higher incidence of *C. abortus*, as the environmental contamination is concentrated in small spaces; conversely in extensively managed flocks, where animals are kept within larger areas, a lower incidence of the pathogen is observed, linked to the fact that animals are less likely to come into contact with a contaminated area [3]. In addition, *C. abortus* can survive in the environment even in unfavourable conditions from a few days to a few months, thanks to the presence of a spore-like cell wall, which gives it considerable resilience [7]. This resistance seems to be directly connected to the greater possibility for the bacterium to come into contact and infect many animal species, farmed or wild ones, and consequently to spread more easily to humans [2]. Specific aspects of this will be discussed later.

To better understand the role of environmental contamination in the spread of the infection we performed a systematic search of the literature available in PubMed and Web of Science databases to retrieve all available studies on *Chlamydia abortus*, including the ones focused on the animal-to-human transmission, using the following search terms: “*Chlamydia abortus*”, “*Chlamydophila abortus* [title]”, “chlamydial abortion [title]”, “*Chlamydia psittaci* (previous nomenclature) [title] abortion [title]”, “chlamydial developmental cycle [title]”, “*Chlamydia psittaci* sheep [title]”, “Enzootic abortion of ewes [title]”, “*Chlamydia psittaci* [title] interferon-gamma”, “Chlamydia cell biology [title]”, “the ovine placenta [title]”, “OEA” [title], “EAE” [title], “Ovine enzootic abortion” and “Enzootic abortion of ewes”. Our review was open to inclusion of studies written in English published in peer-reviewed journals and government reports, selected according to the relevance for the topics covered in the review. Information on genetics and genomics, host–pathogen interactions, infection and pathogenesis of the disease, both for animals and humans, host immune response and the main available vaccines, methods for diagnosis, epidemiology of the disease and environmental aspects are summarized and critically discussed. Our inclusion criteria did not consider any limit in respect of geographical location. All the retrieved studies relevant to the topics were considered suitable for inclusion in the literature review and were cited. Journal articles were not omitted even if only presenting limited data sets. With the keywords previously named the search on PubMed delivered a total of 562 results. Having removed duplicate results and those not available as full text, 180 articles were selected. In addition to the published articles, the books “Intracellular pathogens 1: Chlamydiales” and “Disease of sheep” and the “OIE Terrestrial Manual” were consulted [1, 3, 10–13]. Studies included were variable in terms of topic addressed, experimental design, results and conclusions, and we also incorporated work showing contradictory findings. We extracted information from each study and each of the co-authors reviewed the studies independently before discussing and critically evaluating specific points.

## Genetics and genomics

The zoonotic potential and the possibility of environmental transmission of *Chlamydia abortus* are strictly correlated to the genome type and the genetic diversity of the microorganism. In particular, rearrangements in a variable region of the genome indicated as the “Plasticity Zone” (PZ) and in the tryptophan biosynthetic operon, seem to have evolved to enable better adaptation to the mammalian host and persistence [14]. The genome of *C. abortus* consists of a circular 1.14 million bp chromosome with an overall G + C content of 39.97%. Sequence analysis identified 961 coding sequences (CDS), 746 of which have been functionally assigned, using previously compiled databases, motif matches and experimental evidence. For those with no functional assignments, 110 sequences have been recognized as similar to proteins already found in other members of the *Chlamydiaceae*. Moreover, 29 pseudogenes coding for membrane proteins, *Pmp* (Polymorphic Membrane Proteins)-family proteins and some hypothetical preserved proteins have also been discovered among the 961 CDS. Some functional CDS in the genome of *C. abortus*, appear as pseudogenes in other species within the *Chlamydiaceae* [14]. Complete genomic sequences of several species of *Chlamydia* have been available for some time, such as *C. trachomatis* (2013, GCA_000590575.1), *C. suis* (2018, GCA_900149625.2), *C. psittaci* (2014, GCA_000687395.1) and *C. abortus* (2014, GCA_000952935.1). Comparative analyses between members of the *Chlamydiaceae* family have been published by different groups and highlight the limited correlation between genome conservation and species or tissues tropism. A comparison of the total genome of *C. abortus* with *C. caviae* and *C. pneumoniae* showed a high level of conservation among the three genomes, especially in the region defined as “core”, which was preserved in all three species [14]. This however contrasts with the significant differences shown by these species regarding host range, tissue tropism and disease outcome. Despite the high conservation of the core, several variations were detected, particularly in the “Plasticity Zone” or replication termination region terminus, where the two major clusters of polymorphic genes that encode for TMH (Transmembrane Head)/Inc (Inclusion Membrane Proteins) and *Pmp* proteins and biotin biosynthetic operon are located [14]. The PZ of *C. abortus*, being 12 kb and encoding 11 genes, similarly to *C. pneumoniae*, is very small and with a lower number of CDS compared to *C. caviae*, which encodes for 22 genes. The small size of the PZ of *C. abortus* is mainly the result of loss of the tryptophan biosynthetic operon (trp) [14]. Therefore, *C. abortus* needs to acquire the host’s tryptophan to grow and multiply [15], while this is not required for survival outside the host and for transmission [14]. In the same study, it was suggested that the loss of the trp gene occurred following the niche adaptation of the bacterium [14]. Further adaptation of *C. abortus* can also be seen when comparing the region encoding toxin genes within the PZ of *C. muridarium, C. caviae* and *C. trachomatis* with that of *C. abortus.* These genes have been lost in *C. abortus*, whereas biotin biosynthetic genes, absent in other species, such as *C. caviae*, have instead been identified in its genome [14].

All these differences may reflect the diversity in niche adaptation of the different chlamydial species, thus explaining the variation in host, tissue tropism and pathogenicity that characterizes each species. Once new chlamydial genomes became available, additional comparative analyses might reveal important differences linked to the genomic evolution of these species [8].

## Host–pathogen interaction

The specific interactions between *C. abortus* and its respective hosts have been investigated for some but not all its host species; however, some of the general mechanisms can be extrapolated from work carried out on other *Chlamydia* species [16].

**Figure 1:**
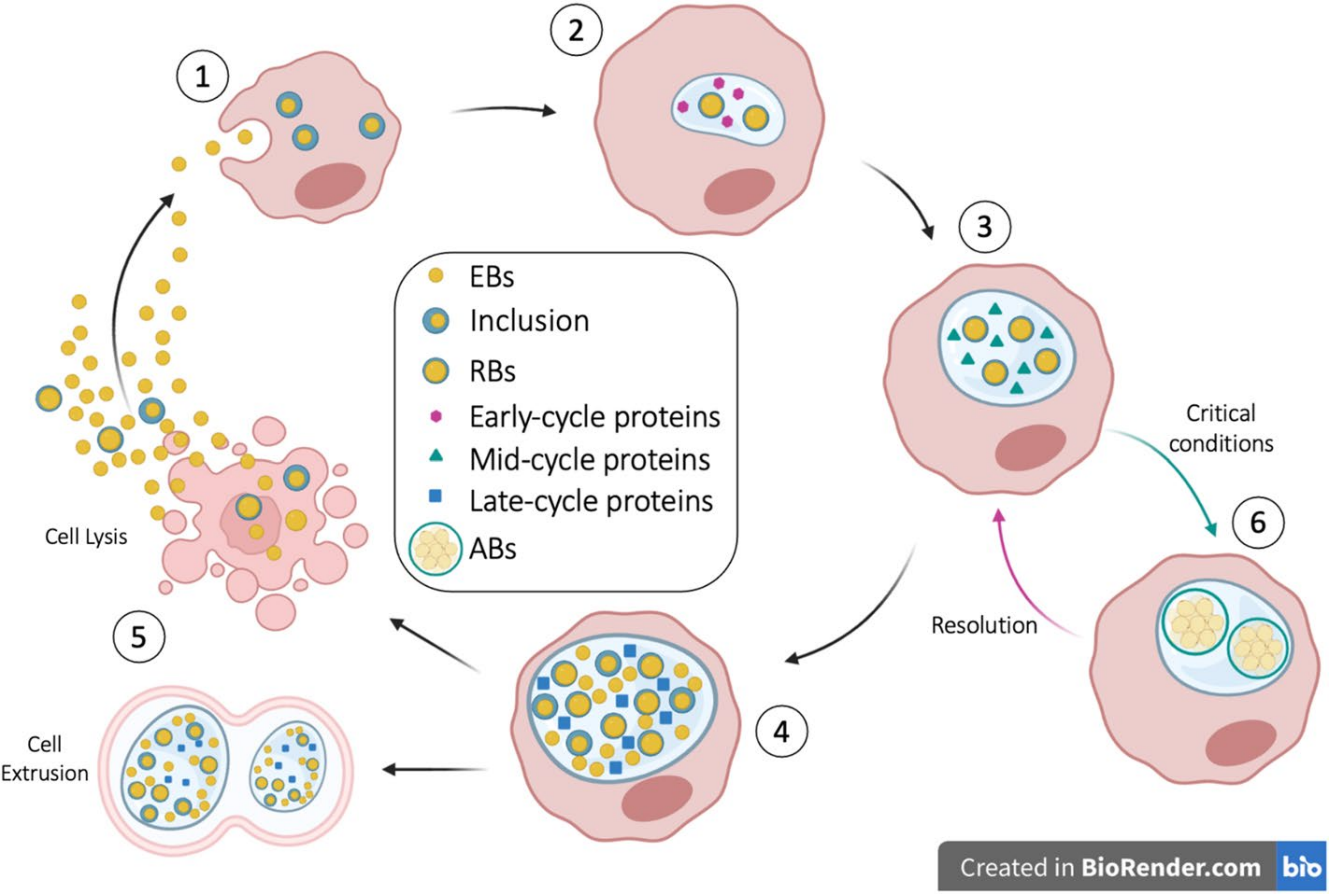
**Schematic representation of** ***Chlamydia*** **developmental cycle.**

The interaction between *Chlamydia* spp. and the host (Figure 1) starts with the infectious form of the pathogen, namely the elementary body (EB) [16] coming in contact with the cell surface, particularly the mucosal epithelial cells. This is followed by cellular adhesion and internalization, and the invasion of the host cell process which relies both on host and bacterial factors [10, 11]. The interaction between bacterial adhesins and *Pmp* proteins (polymorphic membrane proteins) with the host receptor molecules (some yet to be identified) are species-specific, and responsible for host and tissue tropism [11, 17].

Once internalised, *Chlamydia* spp*.* promote the creation of a vacuole known as the inclusion, through actin remodelling [10, 16, 17]. Cell entry and persistence are regulated by a limited number of stress response genes, among which are the genes encoding a chlamydial homologue of GTPase, which is however truncated at the C-terminal domain, therefore unclear if capable to function as true GTPase, but involved in other functions such as cell division, stress response and ribosome maturation [18]. This is followed by the injection of pre-packaged effectors throughout a Type 3 Secretion System (T3SS), able to induce cytoskeletal rearrangements, to promote invasion and to activate host signalling [16].

The EB, now enclosed in a newly formed inclusion, starts a primary differentiation process to become a more conventional bacterial form, the reticulate body (RB), which is also the metabolically active form, which communicates with the host cell through the inclusion membrane, in order to scavenge all the nutrients necessary to grow and multiply [12, 16]. Of central importance, is the capability of *Chlamydia* spp*.* to promote its own fusion with specific cellular compartments, namely the ones rich in nutrients (i.e., exocytic vesicles), while avoiding the fusion with damaging compartments (i.e., lysosomes). The nutrients needed by *Chlamydia* are mainly eukaryotic lipids (i.e., sphingomyelin and cholesterol), necessary for replication, homotypic fusion, growth, reactivation from persistence and for the secondary differentiation from RBs to EBs. Other nutrients, such as amino acids, sugars, nucleotides and fatty acids are likely acquired through the participation of bacterial transporters, porins and translocases [19].

Through the acquisition of nutrients, the bacteria rapidly multiply by binary fission, until the inclusion is completed and enlarged. At this point, following unknown signals, the RB starts a secondary differentiation into EB: in this phase late-cycle genes are expressed, and the newly differentiated EB exits the cell by lysis or by extrusion [16, 17].

RBs can also enter a state of quiescence (persistent state) if exposed to critical environmental conditions, for example the depletion of essential growth substances, or the presence of antibiotics. *C. abortus*, lacking the tryptophan biosynthetic operon (trp), enters into a persistent state if in presence of IFN-γ: this cytokine induces the expression of indoleamine-2,3-dioxygenase (IDO), which in turns degrades the host tryptophan, necessary for bacterial growth. Although *C. abortus* lacks the tryptophan biosynthetic pathway, it can still grow and multiply into the placenta probably through the rescue of tryptophan from maternal-to-foetal nutrients [14]. IDO is known to have multiple functions related to protective immunity (IFN-γ linked), tolerance (by suppression of T cells) and physiology, therefore the animal physiological, nutritional or immune status could influence induction of persistence [20, 21].

## Infection and pathogenesis

In livestock, infection with *C. abortus* occurs mainly via oronasal transmission, after ingestion of the placenta or contaminated bedding or via aerosol inhalation of infectious EBs released in the environment by the infected ewes [15, 19, 22, 23]. Indeed, the disease spreads mostly through environmental contamination from abortion products, in particular placentas and coats of dead/aborted lambs which contain a high bacterial load, and vaginal fluids discharge, which can last up to 10 days post-abortion [9]. The EBs released into the environment are characterized by a great resistance to unfavourable conditions, remaining infectious for a few days after pasture contamination during the spring season, when temperatures are mild, and between few weeks and few months at lower temperatures, when temperatures drop below freezing [9]. This prolonged persistence in the environment poses a serious risk both to farmed and wild animals, which circulate in the contaminated areas, and to people who come into contact with contaminated fields. Once the EBs enter the organism, they are thought to colonise the tonsils and/or the nasopharyngeal lymphatic tissues, and from there disseminate to other organs through blood and lymph [19, 23, 24].

The next stage in the pathogenesis varies according to the physiological status of the animal: if a pregnant ewe is infected up to 5–6 weeks before lambing, the most frequent outcome is a sub-clinical infection manifesting only as clinical disease when abortion (last 2–3 weeks of gestation) or dead/weak lambs appear [3]. If the infection occurs when the ewe is not pregnant or nor in the last 5–6 weeks of gestation [3], no clinical disease or abortion ensues, however a latent form of infection is established (possibly within the lymphoid tissue), and infected ewe will appear healthy until the subsequent pregnancy when abortion can occur [23, 25–27].

The establishment of latency is thought to be related to unfavourable conditions encountered by the bacterium when initially infecting the host. One of the possible causes of latency, as highlighted during in vitro studies, is the presence of IFN-γ, a cytokine produced by the host in response to the infection. IFN-γ is expressed in order to aid the development of a solid immune response against the pathogen, however at the same time the presence of this cytokine causes the instauration of a latent infection, controlled by a continuous low-level release of IFN-γ, in response to antigen pulsing (LPS of *C. abortus*). Since in vivo studies must take in consideration the complex biology of the animal and therefore are more complex to perform, additional knowledge is still required to clarify the persistence mechanism [23, 25]. *C. abortus* then remains in a latent status until the final stages of the sheep pregnancy. At this stage, the bacterium reactivates from latency [25, 28], probably due to pregnancy-related hormonal changes which alter the balance of the immune response: the presence of IFN-γ is harmful for pregnancy, reducing the tolerance to the semi-allogeneic foetus, therefore the immune response shifts from a Th1-type to a Th2-type response. Th2-type cytokines, primarily IL-4 and IL-10, antagonize and downregulate Th1-type cytokines, reducing production of IFN-γ. This reduction in the control mechanism could allow *C. abortus* to multiply and colonize the placenta, causing abortion [25, 29]. In pregnant women however induction of latency is not common, and foetal colonisation is established soon after infection with stillbirth or abortion generally occurring 3 to 8 days after the symptoms start [9]. This is probably related to the different type of placentation, with the sheep synepitheliochorial placenta offering a more robust barrier to the establishment of the infection.

*C. abortus* is able to multiply in several organs, including lungs and liver, however here the infection is controlled by the host [30]. On the contrary, the host fails to control and eliminate the infection in its primary target organ, the placenta [27], particularly in late pregnancy [30]. In sheep, it is only at approximately 60 days of gestation, when maternal haematomata appear in the placentome hilus, that *C. abortus* circulating in the bloodstream or being latent in a yet undefined site is able to begin the actual infection, coming into direct contact with the chorionic epithelium [31, 32]. Work from Álvarez et al. [33] suggests that the bacterium is likely to remain latent on the maternal side of the placenta until the final stages of gestation due to high levels of progesterone, then reactivates in concomitance to the drop of progesterone that occurs at the end of pregnancy, allowing the invasion of trophoblasts and consequently the development of the disease. Although the damages are limited to the hilar zone of some placentomes, the viability and functionality of the surrounding areas are also affected [31]. As well as a placentitis, characterized by chorioallantoic arteritis and thrombosis [34] damage is also found in the foetus, with focal hepatic necrosis and, to a lower extent, small foci of necrosis in lung, spleen, brain and lymph nodes [27]. The underlying cause of abortion is the destruction of the chorionic epithelium and the associated placental damage [9], causing a reduction of the efficiency of the foetal-maternal exchanges [31]. Moreover, the destruction of the chorionic epithelium of the placenta causes an impairment of the hormonal balancing, with a decrease in progesterone levels in the peripheral plasma, with concomitant increase of 17β oestradiol and prostaglandin E2 in the amniotic and allantoid fluids, thus causing premature labour [35–37]. Finally, presence of inflammatory cytokines such as TNF-α and IFN-γ (derived from mononuclear cells in infected arteries and arterioles and in the chorionic epithelium of the infected placentas and from resident monocytes and macrophages, respectively) as well as presence of a mixed inflammatory cell infiltrate with vasculitis and thrombosis in the mesenchyme of the intercotyledonary membranes is also thought to contribute to abortion [5].

After abortion, the contact between the ewe and the highly infected placenta induces a massive antigenic stimulation, followed by instauration of solid immunity, preventing reoccurrence of abortion [25, 38]. Indeed, protective immunity only develops after labour or abortion (or after vaccination) and correlates with seroconversion to specific chlamydial antigens contained in the infected placenta [23].

## Zoonotic infection

*C. abortus* infections in humans are infrequent: in absence of global data, the UK generally reports 1 or 2 cases each per year in pregnant women. The lack of reported cases may be partially explained with the low specificity of the serological tests used to differentiate *C. abortus* from other *Chlamydia* species. Although not frequent, the infection can carry very serious consequences, especially in pregnant women and immunocompromised individuals. The route of infection and the infectious dose for humans are still unclear, but infection is thought to be acquired through contact with infected sheep or goats or secondary contaminated environments and the oronasal mucosa, rather than from incorrect handling of placentas or contact with post-partum secretions by people who work closely with livestock, despite them being generally well aware of the risks [39]. Therefore, the people most at risk of being infected are those who work closely with infected animals, especially during lambing (farmers) and those who work with materials and products derived from infected animals (veterinary surgeons, laboratory technicians, abattoir workers, meet processing plant workers and butchers). Moreover, people who may come into contact with fomites, handling contaminated clothing and boots (hence also the families of the above categories) can also be considered at risk. Finally, those who work with animals recently vaccinated with live attenuated vaccines or who have suffered a vaccine inoculation incident, due to self-injection during vaccine administration are also at increased susceptibility, since the temperature-sensitive attenuated vaccinal strain has shown to be causing disease in some vaccinated animals [40, 41]. *C. abortus* in pregnant women colonizes and replicates in the trophoblast epithelium, causing pelvic inflammatory disease and triggering placental dysfunction and foetal death [42]. The disease presents with a worsening flu-like febrile episode, with fever up to 41 °C [39], associated to thrombocytopenia, coagulopathy, pneumonia and often liver damage [43]. Often the loss of foetus follows closely the initial febrile episode and seriously compromises the health of the mother: septicemia, shock and multi-organ failure can develop, often requiring hospitalisation and intensive care, including mechanical ventilation and catecholamine administration [44]. In non-pregnant women, only one conclusive case has been reported by Walder et al. in 2003 in a 39-year-old woman suffering from chronic abdominal pain, increased vaginal discharge and abnormal menstruation since adolescence. Patient history revealed that at an early age she had been in contact with sheep and contracted *C. abortus* infection as confirmed by *C. abortus* specific PCR and presence of anti-chlamydia antibodies [42]. Borel et al. [45] also investigated a number of patients undergoing intestinal biopsy and found *C. abortus* via DNA microarray analysis in the colon of an 81-year-old female patient; results however were not confirmed by Immunohistochemistry which makes this an intriguing but still unexplained finding. Ortega and collaborators, on the contrary, reported a case of *C. abortus* infection in a 47-year-old male working as a veterinary researcher in a laboratory where studies of intranasal infection of *C. abortus* in sheep were carried out. The patient presented with flu-like symptoms for 7 days and atypical pneumonia, but tested negative for common respiratory pathogens. Serological tests confirmed presence of antibodies specific for *C. abortus* which was also isolated in cell cultures and confirmed by PCR. Sequence analysis confirmed that the strain (*C. abortus* AB7) causing pneumonia was the same strain used in the laboratory. This is the first reported case of human respiratory tract infection by *C. abortus*, and highlights the importance for veterinarians, researchers working in the field and farmers of being vigilant to the possible inhalation of aerosols containing *C. abortus* [46].

## Host immune response and vaccines

Numerous studies have been carried out to elucidate the components involved in the ruminant host immune response, in most cases using murine models, because of the expected similarities in the development of the infection between sheep and mice [47]. Murine models enable the investigation of endometrial tissues (the main target in *C. abortus* infection) since cells that are functionally and morphologically analogue to those present in the endometrial tissues of sheep are also present in mice. In addition, when a pregnant mouse is infected with *C. abortus*, the infection progression and the immune response closely follow the ones observed in the natural host, allowing infection to be eliminated from all organs except the placenta [48]. Consequently, the infection in mice results in a late-term abortion, similar to what happens in infected sheep [7, 47].

Innate immunity is the first line of defence established when a primary infection with *C. abortus* occurs; in addition to controlling the infection, innate immunity is also able to initiate and shape a specific immune response thanks to the secretion of cytokines [48]. Innate immune cells, such as Polymorphonuclear Neutrophils (PMN) and Natural Killer cells (NK) also play a pivotal role [49, 50] as demonstrated by the dramatic influence on the recall of macrophages and T cells (especially CD4^+ve^ and CD8^+ve^) and an increase in mortality following PMN depletion in mouse [49]. Neutrophils-induced recruitment of different cells populations including NK cells, which in turns are able to control the bacterial multiplication, is fundamental in the early control of the infection. Neutrophil depletion is also accompanied by reduction in IFN-γ levels, probably attributable to reduction in NK cell numbers, which correlated with the uncontrolled multiplication of *C. abortus* [50]. Other cellular populations, specifically CD8^+ve^ lymphocytes, are also key components in limiting *C. abortus* infection [51]. In spleen, CD8^+ve^ T cells depletion in experimentally infected mice induced an increase in the secretion of IFN-γ, as well as augmented mortality and morbidity, whereas CD4^+ve^ T cells depletion led to a decrease in morbidity. Therefore, CD8^+ve^ T lymphocytes, in addition in playing an important direct role in controlling the infection, show also regulatory function, namely on the activity of CD4^+ve^ T cells, preventing the immune response from being harmful (immunopathology) to the host [52].

Despite the initial immune response, generally the infection progresses towards the establishment of a persistent infection [53] to eventually reactivate when the sheep host becomes pregnant. This leads in most cases to late abortion of the foetus, while in other cases the delivery occurs normally, although clear placental infection accompanied by pathology can be present. Protective immunity develops at this stage and investigating the mechanisms of this have been at the basis of vaccine development. Vaccination programs triggering a protective immune response and consequent reduction of abortion occurrences can greatly contribute to the reduction in environmental contamination and disease spread. On the contrary, a common farming practice, the use of antibiotics, especially long-acting oxytetracycline (20 mg/kg) during the last month of pregnancy [54] to prevent abortion outbreaks, is not advised. Oxytetracycline treatment is not guaranteed to eradicate disease at flock level, cannot reverse heavy infections and their prophylactic use could potentially lead to emergence of acquired tetracycline resistance, such as that observed for *Chlamydia suis* [55]. However, according to a 1980 study [56], routine administration of oxytetracyclines fortnightly during pregnancy and until lambing seems to reduce or eliminate chlamydial shedding, which is crucial to prevent excretion of *C. abortus* at birth as well as on-farm spread of the infection.

A better preventative approach is instead the use of vaccines. Several vaccines are currently available for the prevention of OEA, depending on the country. Specifically, two live attenuated vaccines are marketed throughout Europe: Enzovax® (MSD Animal Health) and Cevac Chlamydia® (Ceva Animal Health Ltd). In addition, Mydiavac® (Benchmarks Vaccines Ltd. UK) and INMEVA® (Laboratorios Hipra S.A.) [57], both killed vaccines, consisting of inactivated EBs or of their sub-constituents, are also available in the UK and mainland Europe. The live attenuated vaccines are both based on a live attenuated strain, called 1B, which was obtained by chemical mutagenesis of the wild-type strain AB7, making 1B temperature-sensitive and unable to grow at the temperature of 39.5 °C, which is the normal body temperature of sheep [58]. With only ten single-nucleotide polymorphisms (SNPs) differentiating strain 1B from the original wild type strain, it has been suggested that there is no genetic basis for attenuation [41] and vaccine protection has been attributed to the high dose of live microorganisms administered with the vaccine as demonstrated by a recent study where ewes experimentally infected with low (5 × 10^3^ IFU) and medium (5 × 10^5^ IFU) doses of *C. abortus*, developed disease, whereas animals receiving high doses (5 × 10^7^ IFU) showed a protective immune response which prevented abortion [23].

Despite the vaccines conferring a great protection against *C. abortus* and reducing the incidence of abortion, they have some drawbacks: production is expensive, operator infection after accidental injection during administration has been reported, conflicting evidences are outlined on reduction of shedding from ewes and, in some animals, presence of an infection indistinguishable from that seen with the wild-type microorganism, causing the development of placental lesions and abortion in the vaccinated flock [41, 59–64]. On the other side, inactivated vaccines are able to reduce the incidence of abortions and the shedding of *C. abortus* at parturition, but they do not prevent shedding completely [57].

Vaccine research is currently focussed on the development of vaccines that are effective, safe for operators, economical in production and that develop a durable and sterile protective immunity without causing disease or excessive inflammation [53]. The research therefore aims at developing the next generation of vaccines, in particular subunit and DNA vaccines. These are based on a number of different targets including the major outer membrane protein (MOMP), a highly immunogenic protein exposed on the surface [65, 66], CPAF (Chlamydia Protease-like Activity Factor), MIP (Macrophage Infectivity Potentiator) [65] and Pmp18.1 [67]. However, none of these vaccines have yet yielded satisfactory results, lacking effectiveness in protection greater or equal to the live attenuated vaccines currently used [68]. Conversely, vaccine preparations based on *C. abortus* outer membrane complexes (COMC) have shown more promise in several animal models [69–72] as well as in the natural sheep host [73] probably due to the preservation of the native conformation of the different components.

## Diagnosis

An initial diagnosis of Ovine Enzootic Abortion can be achieved by investigating flock history (abortion in the last 2–3 weeks of gestation, birth of stillborn lambs) in combination with examination of the placental membranes which will show inflammation and necrosis [2, 9]. However, diagnostic laboratory confirmation is also required, to differentiate infections by *Coxiella burnetii*, *Campylobacter fetus* and *Toxoplasma gondii* [3]. Diagnosis can be achieved by direct identification of the agent or by serological tests.

Direct identification of the agent can be achieved through staining of smears of placental cotyledons, foetal stomach contents or vaginal swabs of ewes that aborted in the previous 24 h [1] using modified Macchiavello, Giemsa, Brucella differential, or modified Ziehl–Neelsen (MZN) procedures. Of these, MZN is the most reliable method according to the OIE Terrestrial Manual [1, 3]. In order to differentiate *C. burnetii* from *C. abortus*, it is also possible to perform a Fluorescent antibody test (FAT) using monoclonal antibodies or a specific antiserum [1]. Antigen detection with ELISA is also possible, as well as Immunohistochemistry. Diagnosis can also be reached by isolating the agent, through cell cultures, using mainly McCoy or Baby Hamster Kidney (BHK) cells or chicken embryos, starting from infected tissue samples (infected cotyledons, intercotyledonary membranes, vaginal swabs and foetal tissues), but this method is outdated and therefore used only in specific circumstances, such as when direct organism isolation is required [1, 3].

The most frequently used diagnostic methods are however molecular, via conventional or Real Time PCR or DNA microarray/ArrayTube microarray (Sachse et al. 2005). Highly sensitive and specific protocols, targeting the 16S-23S rRNA region or the *pmp* genes have been published by Pantchev et al. (2009) and Laroucau et al. (2001) [75–77]. The PCR can also be combined with restriction fragment length polymorphism (RFLP) analysis, to distinguish between the different *Chlamydia* species, namely *C. psittaci*, *C. abortus* and *C. pecorum* [78, 79]. Real time PCR is widely used for diagnosis because of its specificity, standardized protocols and allows quantification of the chlamydial load [80, 81]. According to the OIE Terrestrial Manual [1] when Real Time PCR is used for diagnosis, a hierarchical use of the technique is recommended, initially performing a PCR screening specific for *Chlamydiaceae*, by amplifying the 23S rRNA region followed by a PCR assay specific to *C. abortus*, targeting sequences of the outer membrane protein [82] or performing a DNA microarray. Another technique allowing a specific and sensitive diagnosis is the TETR (Touchdown Enzyme Time-Release)-PCR assay, which delivers a rapid diagnosis in comparison to classical methods [83]. However, it should be noted that PCR detects only DNA presence and not infectivity; data correlating these two parameters would be very useful, especially when evaluation environmental contamination and transmission events.

Serological tests have been used as diagnostic methods for long time. Historically, the complement fixation test (CF) has been the standard serological method for the diagnosis of OEA, however alternative tests are now preferred, as CF is characterized by low specificity [84], due to cross-reactivity of the LPS antigen, which is present in all *Chlamydiaceae* and in some Gram-negative bacteria (*Acinetobacter*) [1]. Alternatives to the CF are enzyme linked immunosorbent assays (ELISA), which are more sensitive and specific [85, 86]. Many commercial ELISA kits are available, mainly based on three antigens: MOMP, POMP and LPS. Literature comparison between different ELISA assays shows different levels of sensitivity and specificity, for example, an LPS-indirect ELISA proved to be very sensitive, but not specific enough to distinguish between *C. abortus* and *C*. *pecorum* [85]. A MOMP ELISA showed less cross-reactivity when compared to the CF test, although it was shown to be poorly sensitive in most cases [87]. The indirect POMP ELISA on the other hand, showed good sensitivity in many studies and is considered one of the promising alternatives to the CF test [88–90].

Despite the current growth of Environmental DNA approaches for accurate and cost effective detection of pathogens in environmental samples [91], to the authors knowledge, no studies have been conducted to assess *C. abortus* presence and abundance in different environments potentially linked to transmission occurrences. This highlights a research gap which could yield important information for the management and control of the disease and the zoonotic spread.

## Epidemiology

*C. abortus* infection is distributed worldwide, and its disease manifestation, EAE, has been reported in several European countries [92–101] and South and North America [2, 102–106]. There seems however to be a limited number of serological or epidemiological studies carried out in developing countries, such as Asia [6, 107–116] and Africa [117–124]. Furthermore, in New Zealand and Australia, very few cases of infection with *C. abortus* have been identified, and EAE doesn’t seem to represent a major problem [125]. According to the Australian Department of Primary Industries, EAE has never occurred in Australia [126]; however few instances of abortion attributed to infection with *Chlamydia pecorum* in sheep, as well as *C. psttaci* in horses, have been identified in NSW in association with placentitis [127]. West in 2002 and Harvey in 2019 indicated that New Zealand is free of *C. abortus* [127, 128]. Nevertheless, the true prevalence and distribution of EAE however is not completely understood, since relevant data are not collected systematically at a global level and often the only information available is from sporadic reports of diagnosed cases or sero-epidemiological surveys. Figure 2 and Additional file 1 show global seroprevalence rates and detection in different hosts and countries respectively, according to the available (often not exhaustive) data [95, 116, 129–146]. The represented distribution is likely to be underestimated in comparison with the actual prevalence of *C. abortus*, both because of the small amount of data available globally and because of the variable specificity of the tests available for the detection of *C. abortus*.

**Figuer 2:**
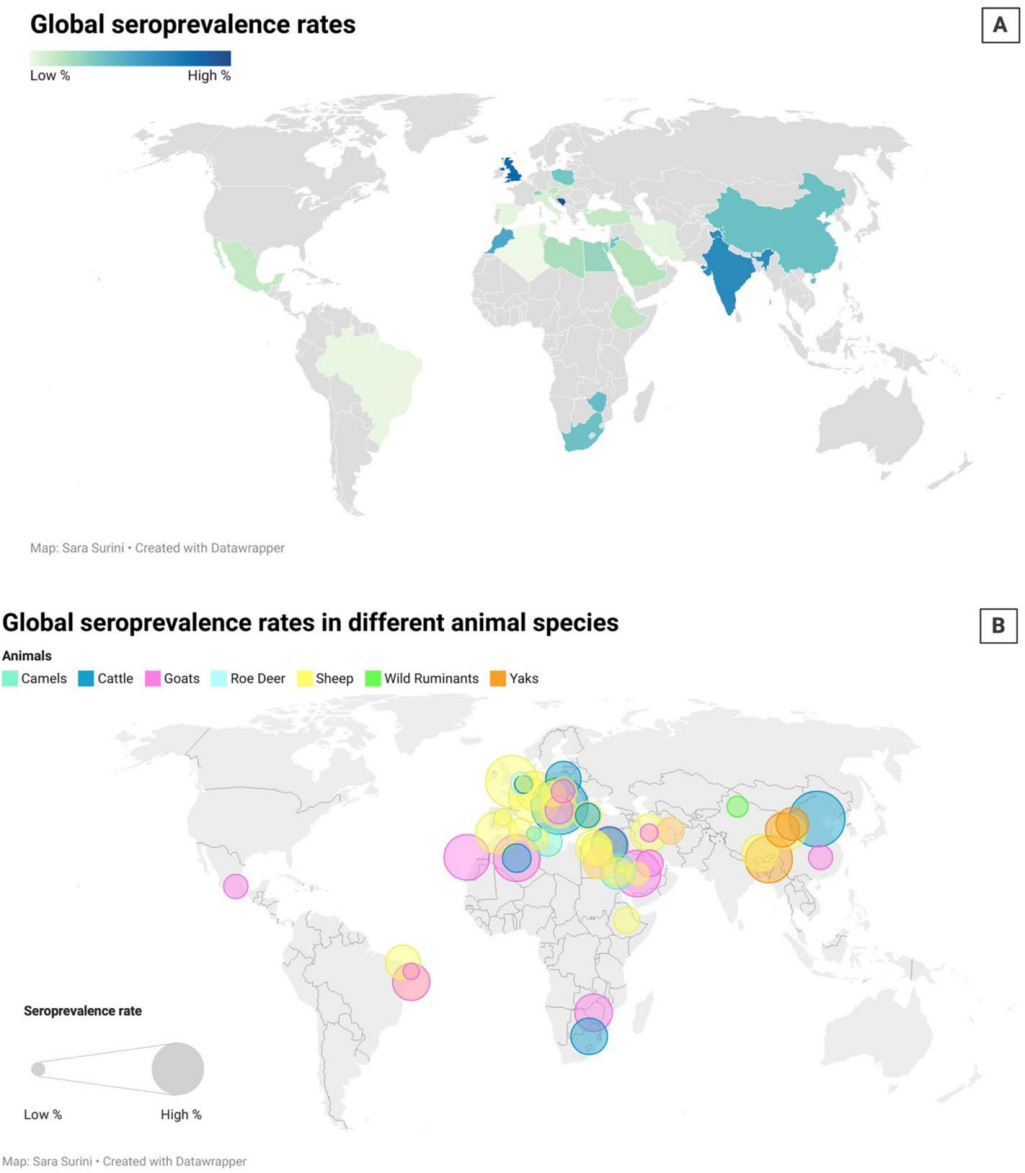
**Representation of the global spread of *C. abortus.*** The reconstruction of the map is based on the collection of data available to date, not exhaustive and probably far from representing the real situation. In addition, the tests used in the seroprevalence study have varying specificity levels, sometimes confusing some Chlamydial species. **A** The percentage of seroprevalence in the different countries where the studies were conducted (different color). **B** The percentage of seroprevalence in the different countries (spot size) and the animal species concerned (different color).

## Environmental contamination

Environment contamination with possibility of transmission of *C. abortus* is generally thought to occur through pasture/farm contamination by placenta, abortion material including dead lambs, the coats of dead and alive lambs and post-partum vaginal secretions. *C. abortus* shows a great resistance to inactivation, mainly due to the EBs structural conformation and the presence of a “outer membrane complex”, a spore-like cell wall stabilized by a series of proteins bound together by disulfide bridges responsible for resistance to the extracellular environment by making the pathogen osmotically stable and poorly permeable [7]. This feature represents a critical point in the transmission of *C. abortus*: following abortion, the EBs-loaded infected placenta is expelled and vaginal discharges are spread in the surrounding environment. These organs and secretions contain the highest bacterial load and represent the main source of infection, both for animals and for humans. In a recent study, aborting ewes were shown to shed higher numbers of *C. abortus* (up to an average of 2.7 × 10^7^ chlamydial genomes per microgram of total tissue DNA) in vaginal swabs and placental material [82] compared to ewes that produced live lambs, when quantified by qPCR [65]; moreover, vaccinated ewes demonstrated even lower levels of bacterial shedding (average 4 × 10^4^).

The risk of transmission through vaginal fluids is reduced 2–3 weeks after abortion, when the discharges begin to dry and stop, although it is still necessary to maintain biosecurity measures [147]. Other risks, although to a lesser extent, are related to the contact of a healthy subject (animal or human) with an aborting sheep, sheep at risk of abortion and dead lambs. An additional risk is related to the ingestion of grass or pasture contaminated by the abortion products by ruminants or other animals [30, 148]. Moreover, Gupta et al. [149] identified presence of *C. abortus* in conjunctival swabs from ruminants, indicating that other organs/apparatuses can also be involved in carrying the bacteria and possibly in interspecies transmission. In pigs, one instance of *C. abortus* identification in conjunctival swab in a potential mixed infection with *C. pecorum* and *C. suis* has been associated with ruminant contact [150]. In horses, occurrences of abortion linked to *C. abortus* infection have been reported [151–153] showing that *C. abortus* is present in equine foetal membranes, therefore these and veterinary or husbandry care around abortion, still birth or foaling should also be considered a potential zoonotic risk.

Seroprevalence studies have further highlighted the wider species distribution of the infection, which also extends to wildfowl [154], psittacids [155], chickens [156], llamas [147], fur animals [157], yaks [158–160], deer [161] and dolphins [162]. In chickens, a study conducted by Szymańska-Czerwińska et al. (2017) identified a seroprevalence of 15.4% at flock level, and this species is thought to represent an asymptomatic carrier [156] which could be relevant for environmental contamination and risk of zoonosis. Serological survey on roe deer (*Capreolus capreolus*) in Flanders by Tavernier et al. in 2015, detected a seroprevalence of *C. abortus* of more than 20% [161] although a similar study in Italian red deer [163] could not find any evidence of exposure. The differences in deer species, the territory covered as well as the probability of coming into contact with livestock might explain this result, or at least evidence the fact that the amount of available information, even based on serosurveys, is still very limited. Farmed fur animals such as foxes, raccoon dogs and mink were also shown, by molecular methods, to harbour *Chlamydia* spp., with *C. abortus* being the dominant *Chlamydia* species identified [157]. Phylogenetic analysis showed high similarity of these *Chlamydia* species to *C. abortus* S26/3 and B577, generally found in ruminants, although there is no information regarding potential for transmissibility between these hosts, other animal species and humans. The authors hypothesise that feed contamination (feed containing *C. abortus*-infected pork), could represent a potential route of transmission, and highlight the potential for a zoonotic transmission through contact with faeces of the infected animals.

Birds, both urban and wild, have also been implicated as possible reservoir species. Urban pigeons were tested for possible transmission of *C. abortus* to humans in a 2012 study [164]: whereas *C. psittaci* was the predominant chlamydial agent detected and cultivated from cloacal swabs and faecal samples, *C. abortus* was also identified in a small number of samples. Testing of community workers in daily contact with the pigeons however did not provide indication of exposure (both PCR and antibody negative). However, in a different study, no *C. abortus* was detected in a large cohort (431) of pigeons and doves, which were instead carrying other chlamydial species [165]. Similarly, an investigation on the potential for outdoor farmed turkeys to harbour and transmit *C. abortus* to abattoir workers provided negative results, albeit detecting 0.7% positivity for another chlamydial pathogen, namely *C. gallinacea* [166]. Oral uptake of *C. abortus* and faecal excretion with or without host infection, could nevertheless contribute to dissemination, as proven by analogy in ducklings where a *C. psittaci* challenge was followed by bird-to-bird transmission of the challenge strains [167], supporting previous findings of grassland contamination through faecal shedding [168]. Moreover, a number of published works in wild birds found chlamydial species that could not be classified but represented instead intermediates between *C. psittaci* and *C. abortus* [169–173]. Recently, a new group of avian *C. abortus* strains with worldwide distribution in various wild bird species has been described [174]. Whole genome sequencing (WGS) of three of these strains (15-70d24, 15-49d3 and 15-58d44, representing genotypes G1, G2 and 1V, respectively) indicated that these avian *C. abortus* strains show features characteristic of both *C. abortus* and *C. psittaci* species, although phylogenetic analyses demonstrated a closer relationship with classic *C. abortus* strains. Currently, species classification established by the ICSP Subcommittee on the taxonomy of *Chlamydiae*, determines that these avian *C. abortus* strains 15-70d24, 15-49d3 and 15-58d44 should be classified as *C. abortus*. However, the authors of this study conclude that the current taxonomic definition of *C. abortus* is outdated and should be amended to include two subgroups, mammalian and avian, the latter of which would include all isolates so far referred to as atypical *C. psittaci* or *C. psittaci*/*C. abortus* intermediates [174]. The zoonotic potential and the potential for environmental contamination from these species is still unknown, however, considering their close relationship to two of the most widespread zoonotic *Chlamydiaceae*, it cannot be excluded.

In Argentina, a study conducted by Origlia et al. in 2019, provided the first description of psittacine pet birds infection in symptomatic and asymptomatic animals. Although this evidence needs to be confirmed by additional studies, a new hypothesis has been formulated regarding the possibility that these animals also represent a reservoir of *C. abortus* [155]. Finally, intermediate *C. psittaci/C. abortus* strains were also isolated from Polish wildfowl [154], as well as swans and mallards which, living in closer contact with human settlements, might also be a possible source of environmental contamination and population exposure. The majority of *Chlamydiae* detected in this study were classified as new genotypes, phylogenetically closer to *C. abortus,* with the authors proposing a reclassification of this species to include isolates of avian origin, as well as mammalian ones.

*C. abortus* has also been detected by molecular methods in a boa constrictor [175] held in captivity, although the significance of this particular finding is not clear, especially considering that snakes harbour *C. pneumoniae*, which is the most commonly reported etiological agent in reptilian chlamydiosis, in addition to their own *Chlamydia* species and several *Chlamydia*-like organisms. Finally, *Chlamydia*-like organisms have been detected in several environments including water [176, 177], inside amoebae [178], in flea [179] and ticks [180, 181].

In Africa, several wild animals from the Serengeti National Park were surveyed by Pospischil et al. (2012) with *C. abortus* identified (by Real Time PCR, microarray and sequencing followed by serological assays) for the first time in African buffalos and a spotted hyena. It is possible that African buffalos may have come into contact with farmed cattle and thus contracted the infection, whereas the positivity of the spotted hyena could be linked to consuming meat of infected animal [182]. In another study carried out in Africa, an increased number of infections in farmed animals was demonstrated in areas where contact with the local wild fauna and people occurs, due to the lack of physical barriers [183]. The same situation seems also to be present in northeastern China in the Xinjiang region, where goitered gazelles (*Gazella subgutturosa*) were found positive for *C. abortus*, and thus are considered a carrier in this region [184].

Overall, our literature search revealed lack of data on environmental detection, distribution and quantification of *C. abortus*. Therefore, further work should address these gaps to evaluate the degree of contamination of pastures and other environmental areas potentially infected by *C. abortus* in relation with conditions such as temperature and humidity, in order to establish exposure risks. By analogy, *C. psittaci* elementary bodies can remain infectious in the environment for months with persistence on dry inanimate surfaces reported for up to 15 days [185]. Another challenge is represented by the identification of the geographic distribution of *C. abortus* among wild and farmed animals.

## Management of disease

Following an initial abortion a number of approaches are required to limit environmental contamination and infection spread until a definitive diagnosis is reached. Ewes that have aborted must be isolated for a minimum of 7–10 days, or until the post-partum discharges have dried up. Live lambs, if present, must also be isolated with their mother. Bedding, placenta and dead lambs must be disposed safely, using protective equipment (gloves and waterproofs at minimum) and limiting contact with farm staff and other individuals to the minimum possible [2, 3, 186]. Cleaning and disinfection of the affected area needs to be carried out as soon as possible, again wearing PPE and washing hands after contact with the affected animals and possible fomites. PPE and work clothes need to be washed and/or disinfected as soon as possible, considering the possibility of transmission of the infection to other members of the household. *C. abortus* is resistant to acid and alkali therefore disinfectants such as quaternary ammonium compounds, isopropanol alcohol, household bleach or chlorophenols are recommended for cleaning potentially infected areas. Following the initial abortion episode, it is important to identify additional infected ewes as soon as possible to limit the spread of the infection, not only during the current lambing seasons but also to minimise consequences in the next breeding season. Despite being refractory to another abortion episode, ewes that have aborted might became persistently infected and excrete *C. abortus* at the next breeding season [2, 3, 186]. Moreover, ewes or lambs that became infected as consequence of the initial abortion can became latently infected and abort at the successive season. Therefore, a close flock, or one where replacement animals are acquired from an accredited breeder, along with vaccination, is the optimal way to reduce the introduction of *C. abortus* in a flock. Additionally, rams, birds, dogs, feral sheep and wildlife should be considered for potential zoonotic risk [154, 157].

## Conclusions

This review encompasses the available research findings related to *C. abortus* and highlights future research needs, which are mainly related to the risk of exposure to this microorganism. Besides farmed animals, wild or non-farmed animals can act as reservoirs, and therefore can contribute to the transmission and environmental spread of *C. abortus* in different locations, both to other animals and humans, making controlling the spread of the pathogen more difficult. In this regard, all the evidence indicate that future work should concentrate on multiple topics, including the deepening of bacterial evolutionary strategies leading to genetic and genomic changes responsible for host and environment adaptation, the elucidation of host–pathogen interaction mechanisms and some pathogenetic aspects linked to transmission, latency, host immune response and persistence. More complete epidemiological surveys at animal and human levels are also advisable in order to determine distribution and quantification of *Chlamydia abortus* worldwide, as well as the development of protective and safe new generation vaccines. For example, a number of studies conducted on non-canonical species (eg avian, wildlife, reptiles etc.) has also provided contradictory findings, with contrasting seroprevalence levels in similar species observed and/or presence/absence detected at molecular level. Whereas differences in cohorts, methodologies and population dynamics can explain this observation, this highlights the need for more work in this area to understand which additional animal species can act as carriers and their relative importance to the spread of the disease to livestock and humans.

Finally, there is an almost complete lack of evidence related to environmental diagnosis, along with the need for the improvement of serological tests and the definition of correlations between infectivity and molecular detection. Further knowledge of the impact of available vaccines on environmental contamination as well as the development of candidate vaccines able to control the bacterial shedding also represent an urgent need.

Addressing these research needs will improve the knowledge, assess the extent of the problem, and consequently develop more effective control strategies to reduce *C. abortus* transmission between animals, wildlife and humans within an “One Health” approach.

## Supplementary Information

**Additional file 1.** **Epidemiological studies reporting animal species and prevalence rate of global spread of *Chlamydia abortus*** **according to the currently available data.** The represented distribution is likely to be underestimated in comparison with the actual prevalence of *C. abortus*, both because of the small amount of data available globally and because of the variable specificity of the tests available for the detection of *C. abortus*.

## Abbreviations

*C. abortus*: *Chlamydia abortus*
EAE: enzootic abortion of ewes
OEA: ovine enzootic abortion
CDS: coding sequence
*pmp*: Polymorphic membrane proteins
TMH/Inc: transmembrane head/Inclusion membrane protein
PZ: plasticity zone
trp: tryptophan
EB: elementary body
T3SS: Type 3 secretion system
RB: reticulate body
IFN-γ: interferon-gamma
IDO: indoleamine-2,3-dioxygenase
LPS: lipopolysaccharide
Th1: T helper 1
Th2: T helper 2
IL: interleukin
TNF-α: tumor necrosis factor-alpha
PCR: polymerase chain reaction
PMN: polymorhonuclear
NK: natural killer
SNP: single-nucleotide polymorphism
MOMP: major outer membrane protein
CPAF: Chlamydia protease-like activity factor
MIP: macrophage infectivity potentiator
COMC: *Chlamydia abortus* outer membrane complex
MZN: modified Ziehl–Neelsen
FAT: fluorescent antibody test
BHK: Baby Hamster Kidney
RFLP: restriction fragment length polymorphism
TETR: touchdown enzyme time-release
CF: complement fixation
ELISA: enzyme linked immunosorbent assay
POMP: polymorphic outer membrane protein
NSW: New South Wales
qPCR: quantitative PCR
PPE: personal protective equipment
