# Supplementary material for: Recent advances and public health implications for environmental exposure to Chlamydia abortus: from enzootic to zoonotic disease
Source: Vet Res. 2022 May 31;53:37. doi: 10.1186/s13567-022-01052-x (PMC9152823; doi:10.1186/s13567-022-01052-x)
Supplement: Supplementary file 1 — Additional file 1. Epidemiological studies reporting animal species and prevalence rate of global spread of Chlamydia abortus according to the currently available data. The represented distribution is likely to be underestimated in comparison with the actual prevalence of C. abortus, both because of the small amount of data available globally and because of the variable specificity of the tests available for the detection of C. abortus. [file 13567_2022_1052_MOESM1_ESM.docx]

| PLACE | ANIMAL SPECIES | PREVALENCE RATE | REFERENCE |
| --- | --- | --- | --- |
| Algeria | Cattle | 12.20% | 119 |
| Algeria | Goats | 35% | 129 |
| Algeria (Eastern) | Camels | 2.50% | 124 |
| Algeria (Northeastern) | Sheep | 7.20% | 118 |
| Austria (Vorarlberg) | Sheep | 9.20% | 130 |
| Belgium (Flanders) | Roe Deer | 6.70% | 163 |
| Belgium (Limburg) | Sheep | 4.05% | 131 |
| Belgium (Walloon Brabant) | Cattle | 4.23% |  |
| Bosnia and Herzegovina | Cattle | 52.10% | 98 |
| Brazil (Alagoas) | Sheep | 21.50% | 104 |
| Brazil (Alagoas) | Goats | 21.50% | 104 |
| Brazil (Cearà) | Sheep | 18.45% | 132 |
| Brazil (Rio Grande do Norte) | Goats | 3.5% | 133 |
| China | Cattle | 50.10% | 112 |
| China (Gansu Province) | Sheep | 18.65% | 111 |
| China (Hunan Province) | Goats | 8.45% | 114 |
| China (Qinghai Province) | Yaks | 17.66% | 160 |
| China (Tibet) | Sheep | 20.90% | 134 |
| China (Xinjiang) | Wild Ruminants | 6.20% | 186 |
| China (Zhuaxixiulong, Xidatan) | Yaks | 16.22% | 161 |
| Croatia | Sheep | 20% | 135 |
| Croatia | Goats | 11.40% |  |
| Egypt | Goats | 14.90% | 136 |
| Egypt | Sheep | 14.90% |  |
| Egypt (Alexandria) | Sheep | 19.20% | 123 |
| Egypt (Kafr Elsheik) | Sheep | 15% |  |
| Ethiopia | Sheep | 9.88% | 121 |
| India (Arunachal Pradesh) | Yaks | 35% | 108 |
| Iran | Sheep | 21.70% | 137 |
| Iran | Goats | 4.30% |  |
| Iran (Khorasan Razavi province) | Sheep | 9.70% | 115 |
| Iran (Khorasan Razavi province) | Goats | 10.20% |  |
| Italy | Sheep | 4.80% | 93 |
| Italy | Goats | 5.80% |  |
| Jordan | Sheep | 21.80% | 138 |
| Jordan | Goats | 21.80% |  |
| Jordan | Cattle | 19.90% | 109 |
| Libya | Camels | 12.20% | 139 |
| Mexico | Goats | 8.50% | 102 |
| Morocco | Sheep | 27.20% | 140 |
| Egypt (Northern) | Sheep | 13.70% | 123 |
| Poland | Cattle | 19.30% | 141 |
| Saudi Arabia | Camels | 19.40% | 142 |
| Saudi Arabia | Sheep | 11.10% | 116 |
| Saudi Arabia (Eastern) | Goats | 10.60% | 116 |
| Saudi Arabia (Medina) | Sheep | 0.75% | 143 |
| Saudi Arabia (Riyadh) | Sheep | 7.52% | 144 |
| Saudi Arabia (Riyadh) | Goats | 34.50% |  |
| Slovak Republic | Sheep | 11.70% | 94 |
| Slovak Republic | Goats | 7.70% |  |
| South Africa | Cattle | 20.70% | 145 |
| Spain | Sheep | 3.90% | 146 |
| Spain (canary island) | Goats | 33% | 99 |
| Switzerland | Sheep | 15.5% | 147 |
| Switzerland (Graubünden) | Sheep | 42% |  |
| Tunisia | Sheep | 6.60% | 120 |
| Turkey | Sheep | 11.80% | 148 |
| Turkey | Cattle | 8.70% |  |
| United Kingdom | Sheep | 44% | 123 |
| Zimbabwe | Goats | 22% | 185 |
